# Upper extremity motor abilities and cognitive capability mediate the causal dependency between somatosensory capability and daily function in stroke individuals

**DOI:** 10.1038/s41598-021-04491-2

**Published:** 2022-01-13

**Authors:** Szu-Hung Lin, Tong-Rong Yang, I-Ching Chuang, Chia-Ling Chen, Ching-Yi Wu

**Affiliations:** 1 Department of Psychology, Soochow University, Taipei, Taiwan; 2 Department of Psychology, National Taiwan University, Taipei, Taiwan; 3 Department of Occupational Therapy& Graduate Institute of Behavioral Science, College of Medicine, Chang Gung University, No. 259, Wenhua 1st Rd., Guishan Dist., Taoyuan City, 33302 Taiwan; 4 Department of Neurology, Chang Gung Memorial Hospital, Linkou, Taoyuan Taiwan; 5 Department of Physical Medicine and Rehabilitation, Chang Gung Memorial Hospital, Linkou, Taiwan; 6 Graduate Institute of Early Intervention, Chang Gung University, Taoyuan, Taiwan; 7 Healthy Aging Research Center, Chang Gung University, Taoyuan, Taiwan

**Keywords:** Diseases, Stroke

## Abstract

Stroke individuals’ daily function has been demonstrated to be influenced by their somatosensory capability, cognitive capability, and upper extremity (UE) motor abilities. However, the structural relationships among these abilities on stroke individuals’ independence in daily function remain unclear. We analyzed the pretest measures of 153 stroke individuals in outpatient rehabilitation settings by structural equation modeling to determine the structural relationship among somatosensory capability, UE muscle strength, UE motor function, and cognitive capability that influences independence in daily function. The standardized results indicated somatosensory capability negatively influenced UE muscle strength, but positively influenced UE muscle strength mediated by UE motor function. UE muscle strength, then, positively influenced individuals’ independence in daily function. On the other hand, somatosensory capability positively influenced cognitive capability, which marginally and positively affected the performance of independence in daily function. To the best of our knowledge, this is the first study to demonstrate the influence of somatosensory capability on the daily function is mediated mainly by motor functions and marginally by cognitive capability. This structural model may allow future clinical therapists to design more effective task-related training protocols to promote the independence in daily function for stroke individuals.

## Introduction

Most stroke individuals suffer from long-term disabilities in various domains, such as somatosensory^1^, motor^2^, and cognitive deficits^3^. Several studies have indicated that these deficits affect the functional independence of performing the activities of daily living (ADL). Rehabilitation is important to help stroke individuals regain maximal ability for independently performing ADLs. To provide an effective rehabilitation protocol to restore a stroke individual’s functional independence, recognition of the relationships between these deficits and the stroke individual’s independence in daily function is fundamental.

Among these deficits, motor impairment, especially in the upper extremity (UE), is encountered by 50% to 80% of patients in the acute phase^4,5^, which usually leads to complete or partial dependence for the ADL. Previous studies have identified several components of motor abilities that are associated with the ADL performance; for example, Bae et al.^6^ investigated the relationship between grip and pinch strength and independence in ADL in chronic stroke individuals, and found that patients’ UE muscle strength of the affected hand was associated with independence in ADL. In addition, Franceschini et al.^7^ identified that the UE motor function assessed by the Box and Block Test (BBT) is the most robust predictor of the performance of ADLs after robot-assisted training.

Well-coordinated movements for successfully performing the task contribute to the recovery of UE muscle strength^8^. Bütefisch et al.^9^ demonstrated that repetitively performing identical hand and finger movements is beneficial for improving grip strength. Several therapeutic approaches to motor rehabilitation of patients with UE impairment have been established aiming to restore stroke individuals’ functional independence of the ADL. Thus, examining the causal relationship among different components of the motor function and the ADL would be useful for therapists to design an effective rehabilitation protocol.

Somatosensory deficit is one of the most common complications of stroke individuals^1^ and has also been reported to be a significant link to the performance of the ADL^10,11^. Accumulated evidence found that the restoration of the somatosensory capability (i.e., proprioception and exteroceptive sensation) has positive impact on the improvement of UE motor function^12,13^ and the ADL in stroke individuals. For example, somatosensory disturbance may be the crucial factor in poor hand function because functional hand use requires not only motor function and dexterity but also tactile and haptic input to modulate and help motor command generation. Proprioception deficits are important predictors of poor functional outcome after stroke, and the tactile deficit would be one of the multiple perceptual problems in determining functional recovery from stroke individuals^14^. In addition, a previous study provided a hierarchical pathway from somatosensory processing to cognitive processing and higher-order cognitive function by using structural equation model (SEM) technique^15^. Stroke individuals generally have universal^16^ and persistent cognitive deficits^17,18^ and significant correlations of various components of the ADL or instrumental ADL (IADL) to one or many particular cognitive components^19,20^. Improving stroke individuals’ somatosensory and cognitive capability are 2 of the top priorities in various rehabilitation programs.

Although previous studies have investigated the association of each deficit and ADL/IADL performance, no study to date has attempted to clarify the structural relationship of these common deficits and the ADL. The covariance-based SEM technique would be a useful method to examine the structural relationships among these measures. SEM is a technique used for specifying and estimating linear relationships among different aspects of measures, and these measures are theorized to be related to one another with a structure^21,22^. The structure implies statistical and often causal relationships between measures, that is, a hypothesized pattern of linear relationships among these measures.

By using cross-sectional data from motor rehabilitation trials and the SEM technique, we aimed to clarify the causal dependencies among somatosensory capability, UE motor abilities (UE muscle strength and UE motor function), cognitive capability, and ADL in stroke individuals. In addition, while stroke individuals typically achieve independence in ADLs, they may require additional supports when returning to work or leisure activities or have problems with carrying out IADLs^23^. ADLs involve those very basic activities that individuals perform in their everyday life, such as eating, bathing and using the bathroom^24^. IADLs are complex tasks and built on those basic ADLs, which focus on helping an individual flourish in the community and become completely self-reliant in his or her daily life. Examples of IADLs include shopping, housekeeping (or housework), accounting (or managing money), food preparation for family and transportation^24^. Thus, in this study, we used the measurement of IADL to indicate the independence in daily function.

Previous studies showed that somatosensory capability has impact on the UE motor function^12,13^ and cognitive capability^15^, and UE motor function and cognitive capability are associated with the independence of the ADL^6,7,19,20^. We hypothesized that somatosensory capability would directly influence cognitive capability, UE muscle strength, and UE motor function and that these latent constructs would directly influence the independence in daily function. Based on the evidence that recovery and repetition of motor execution may contribute to build UE muscle strength^8,9^, we further hypothesized that UE motor function would also influence the UE muscle strength, and then the UE muscle strength influence the independence in daily function. That is, the transmission of the influence of UE motor function on independence in daily function might be through UE muscle strength.

An examination of how these deficits interplay to predict the performance of the independence in daily function in stroke individuals would enhance our understanding of the mechanisms between these deficits and the patient’s ability to perform the daily function and might shed light on the possible directions for providing personalized and hierarchical treatment protocols for stroke individuals. Specifically, understanding the predictive relationship of these deficits to individual’s performance of independence in daily function is important for us to set realistic treatment goals and to know what aspects may be strengthened in future trials to enhance the transfer of improved sensitive, cognitive, and UE motor abilities in daily life.

## Methods

### Participants

This study used pretest data from previous and ongoing trials of stroke rehabilitation therapy conducted at 2 medical centers, 8 regional hospitals, 2 district hospitals, and 1 clinic in Taiwan. Included were stroke individuals who completed the behavioral assessments before the intervention program. Informed written consent was obtained by all the participants. The execution of these studies and the informed consent of these studies were approved by the Institutional Review Board of Chang Gung Memorial Hospital, Taipei Medical University, Cheng Hsin General Hospital, China Medical University & Hospital, Cathay General Hospital, Taipei Hospital of Ministry of Health and Welfare, and the Buddhist Tzu Chi General Hospital. All methods were carried out in accordance with relevant guidelines and regulations.

The general inclusion criteria for participants were (1) sustained a unilateral stroke with onset ≥ 3 months, (2) modified Ashworth Scale score ≤ 3, (3) UE Fugl-Meyer assessment score between 18 and 57, indicating mild to moderate mild motor severity, and (4) age ≥ 18 years. The general exclusion criteria were (1) additional neurologic or psychologic disorders other than stroke (e.g., Parkinson disease), (2) receipt of *Botulinum* toxin injections administered 3 months before enrollment, or (3) unable to follow instructions and perform the tasks (Mini-Mental Status Examination [MMSE] score ≤ 20).

### Outcome measures

#### Measures of somatosensory capability

The Revised Nottingham Sensory Assessment (rNSA) was used to evaluate the sensation of stroke individuals, and its psychometric properties have been established^25^. It consists of 72 items grouped into three subscales measuring tactile sensation, proprioception, and stereognosis. The Tactile Sensation subscale includes light touch, temperature, pinprick, pressure, tactile localization, and bilateral simultaneous touch and is administered to the face, trunk, shoulder, elbow, wrist, hand, knee, ankle, and foot. The rNSA is scored on a 3-point ordinal scale (0–2), with a lower score suggesting greater somatosensory impairment.

#### Measures of UE muscle strength

The Medical Research Council scale (MRC) is an ordinal scale that assesses the UE muscle strength in stroke individuals, and the reliability of this measurement is good to excellent^26^. This scale measures wrist extension, finger extension, elbow flexion and shoulder abduction on both sides. The scoring for each muscle ranges from 0 to 5, with a higher score indicating stronger muscle.

#### Measures of UE motor function

The Wolf Motor Function Test (WMFT) and the Chedoke Arm and Hand Activity Inventory (CAHAI) were used to assess the functional ability of the stroke individual’s UE. The WMFT was developed by Wolf and colleagues to quantitatively assess UE motor function^27^. The WMFT-time measures the time required to complete the tasks, and the WMFT-quality assesses functional ability on a 6-point ordinal scale. A lower WMFT-time performance indicates faster movement, and a higher WMFT-quality score suggests better quality of movement. The reliability of the WMFT is excellent^28^.

The CAHAI is used to evaluate the functional ability of the paretic UE to perform tasks. It uses a 7-point quantitative scale to measure the client’s ability to complete the task using their bilateral hand. A higher score indicates that patients have better ability to complete the functional movement independently. The reliability and the internal consistency of the CAHAI is good in individuals with stroke^29^.

#### Measures of general cognitive capability

The Montreal Cognitive Assessment (MoCA) was used as a general measurement of cognitive capability in this study. This assessment tool is a 30-point test^30^. The MoCA has been recommended as a valid and reliable clinical assessment in stroke individuals^31^.

#### Measures of the independence in daily function

The Nottingham Extended Activities of Daily Living Scale (NEADL) is a self-report scale that measures IADL. It evaluates 4 areas of daily function, including mobility, kitchen, domestic, and leisure activities. The total score is 66, and a higher score indicates better daily functional ability. The psychometric properties of the NEADL have been well established^32^.

### Statistical analysis

The statistical analyses for this study were generated using SAS/STAT 9.4 software with the CALIS procedure (SAS Institute Inc, Cary, NC). A *P* value of ≤ 0.05 was considered to indicate statistical significance. The participants’ demographic characteristics are summarized with descriptive statistics.

To examine the structural relationships among these measures, we used the covariance-based SEM technique, which is a multivariate statistical analysis technique for representing, estimating, and testing a theoretical network of linear relationships among a set of measured variables and latent constructs^21^. In this study, the construct of *somatosensory capability* was measured by the 3-item parcels of the rNSA (tactile sensation, proprioception, and stereognosis). The construct of the *UE muscle strength* was measured by the 4-item parcels of the MRC. The construct of *UE motor function* was evaluated by the assessments of the WMFT time, the WMFT quality, and the CAHAI score. The 7-item parcels of the MoCA indicated the construct of *cognitive capability*. Finally, the 4-item parcels of the NEADL were used to indicate the construct of *independence in daily function*. In this study, although several ordinal measured variables (such as rNSA, CAHAI, and NEADL) were used, these ordered categorical data have at least five categories so that they could be treated as continuous variables in using SEM^33,34^.

The confirmatory factor analysis (CFA) was used to evaluate the goodness of fit of the measurement model containing the 5 latent constructs (factors) and 21 measured variables. To examine the fit of the structural model, the directional relationship of the latent constructs to the model of independence in daily function was determined by SEM. Maximum likelihood estimation was used to estimate factor loading, path coefficient, and the amount of measurement error.

No single model-fitting index can consider all scenarios^35^, so we used indicators with different characteristics to evaluate the goodness of fit of the hypothesized model. According to recommendations of previous research^36,37^, the fit of the measurement and structural model was assessed with the *P* value of the χ^2^ statistic > 0.05 (*P* > 0.05), χ^2^/degree of freedom (df) of < 2, comparative fit index (CFI) of > 0.95, root mean square error of approximation (RMSEA) of < 0.05, and standardized root mean residual (SRMR) of < 0.08.

Considering the theoretical and empirical significance of the associations among these latent constructs, the entire model was revised by the suggestion of the multivariate Lagrange Multiplier (LM) and Wald test to improve the goodness of fit of the model^38^. The multivariate LM test identifies parameters that would lead to the largest drop in the model χ^2^ and calculates the expected change in χ^2^, thus we can add these parameters to the model (i.e., added a covariance between error terms of variables) to improve the model fitting. The Wald test is analogous to backward deletion of variables in stepwise regression, where one seeks a nonsignificant change in *R*^2^ when variables are left out.

## Results

This study collected the evaluation data of 153 stroke individuals (109 men), with a mean age of 56.19 ± 11.71 years. Table 1 summarizes the demographic and clinical characteristics as well as evaluation data of the participants. Table 2 shows the association between observed variables.

**Table 1:** Demographic and clinical characteristics, and results of outcome measures (*N* = 153).

Characteristics	*n* (%) or Mean ± SD
Age (years)	56.19 ± 11.71
Male (%)	109 (71.2%)
Time after stroke (months)	28.03 ± 24.82
Education (years)	11.31 ± 4.41
MMSE	28.13 ± 1.99
FMA-UE	34.55 ± 9.75

Outcome measures	Mean ± SD
**Somatosensory capability (rNSA)**	
Tactile	75.16 ± 28.39
proprioception	16.82 ± 5.22
Stereognosis	13.65 ± 8.68
**Cognitive capability (MoCA)**	
Total score	24.17 ± 4.22
Visuospatial	3.75 ± 1.18
Naming	2.78 ± 0.55
Attention	5.25 ± 1.06
Language	2.1 ± 1.01
Abstract	1.35 ± 0.76
Recall	3.19 ± 1.6
Orientation	5.75 ± 0.55
**UE motor function (WMFT & CAHAI)**	
WMFT_time (seconds)	11.14 ± 5.61
WMFT_quality	2.66 ± 0.61
CAHAI	41.18 ± 17.80
**UE muscle strength (MRC)**	
Shoulder	3.42 ± 1.13
Elbow	3.94 ± 1.06
Wrist	2.48 ± 1.2
Finger	2.67 ± 0.98
**Independence in daily function (NEADL)**	
Mobility	12.14 ± 4.46
Kitchen	5.84 ± 4.49
Living	5.38 ± 4.44
Leisure	7.61 ± 3.82

MMSE: Mini-Mental State Examination; FMA-UE: Fugl-Meyer Assessment for upper extremity; rNSA: Revised Nottingham Sensory Assessment; MoCA: Montreal Cognitive Assessment; WMFT: Wolf Motor Function Test; CAHAI: Chedoke Arm and the Hand Activity Inventory; MRC: Medical Research Council scale; NEADL: Nottingham Extended Activities of Daily Living Scale.

**Table 2:** Pearson’s correlation coefficients (*r*) among observed variables (*N* = 153).

	Somatosensory capability			Cognitive capability							UE motor function			UE muscle strength				Independence in daily function			
	S1	S2	S3	C1	C2	C3	C4	C5	C6	C7	F1	F2	F3	M1	M2	M3	M4	D1	D2	D3	D4
**Somatosensory capability (rNSA)**																					
Tactile (S1)	1.00	0.84**	0.72**	0.21*	0.06	0.26*	0.14^†^	0.06	0.09	0.14^†^	− 0.3**	0.28**	0.26*	0.03	0.03	0.13	0.04	0.10	0.12	0.04	0.17*
Proprioception (S2)		1.00	0.7**	0.19*	0.07	0.25*	0.15^†^	0.07	0.02	0.13	− 0.3**	0.32**	0.28**	0.05	0.08	0.17*	0.04	0.07	0.12	0.01	0.11
Stereognosis (S3)			1.00	0.16^†^	0.14^†^	0.29**	0.23*	0.13	0.08	0.09	− 0.29**	0.35**	0.32**	0.1	0.09	0.21*	0.1	0.00	0.03	0.02	0.14^†^
**Cognitive capability (MoCA)**																					
Visuospatial (C1)				1.00	0.30**	0.45**	0.28**	0.26*	0.30**	0.03	− 0.05	− 0.04	0.07	0.15^†^	0.06	0.04	− 0.09	0.11	0.09	0.12	0.22*
Naming (C2)					1.00	0.32**	0.42**	0.37**	0.25*	0.13	0.03	0.04	0.05	0.04	− 0.02	− 0.01	− 0.01	0.08	0.02	0.18*	0.24*
Attention (C3)						1.00	0.45**	0.36**	0.29**	0.15^†^	0.09	− 0.03	0.01	− 0.02	− 0.16*	− 0.06	− 0.19*	0.09	0.07	0.1	0.26*
Language (C4)							1.00	0.40**	0.27**	0.27**	0.01	0.14^†^	0.14^†^	0.21*	0.09	0.04	0.07	0.05	0.08	0.09	0.37**
Abstract (C5)								1.00	0.18*	0.06	0.14^†^	− 0.05	− 0.08	0.04	− 0.06	− 0.08	0.02	0.02	− 0.01	0.02	0.32**
Recall (C6)									1.00	0.08	0.03	0.02	0.08	0.17*	0.03	0.03	0.04	0.16*	0.08	0.12	0.23*
Orientation (C7)										1.00	− 0.03	0.19*	0.10	− 0.02	0.08	0.13	0.08	0.08	0.11	0.08	0.19*
**Functional use (WMFT & CAHAI)**																					
WMFT_time (F1)											1.00	− 0.67**	− 0.56**	− 0.20*	− 0.24*	− 0.54**	− 0.45**	− 0.15^†^	− 0.21*	− 0.09	0.06
WMFT_quality (F2)												1.00	0.78**	0.30**	0.40**	0.65**	0.66**	0.16*	0.20*	0.11	− 0.07
CAHAI (F3)													1.00	0.27**	0.34**	0.52**	0.55**	0.08	0.15^†^	0.11	− 0.05
**UE muscle strength (MRC)**																					
Shoulder (M1)														1.00	0.50**	0.20*	0.37**	0.19*	0.14^†^	0.12	0.12
Elbow (M2)															1.00	0.40**	0.40**	0.20*	0.16*	0.13	0.00
Wrist (M3)																1.00	0.66**	0.14^†^	0.18*	0.10	− 0.12
Finger (M4)																	1.00	0.12	0.17*	0.10	− 0.09
**Independence in daily function (NEADL)**																					
Mobility (D1)																		1.00	0.61**	0.63**	0.31**
Kitchen (D2)																			1.00	0.70**	0.25*
Living (D3)																				1.00	0.36**
Leisure (D4)																					1.00

MMSE: Mini-Mental State Examination; FMA-UE: Fugl-Meyer Assessment for upper extremity; rNSA: Revised Nottingham Sensory Assessment; MoCA: Montreal Cognitive Assessment; WMFT: Wolf Motor Function Test; CAHAI: Chedoke Arm and the Hand Activity Inventory; MRC: Medical Research Council scale; NEADL: Nottingham Extended Activities of Daily Living Scale.

^†^*P* < 0.10; *P < 0.05; **P < 0.001.

### CFA of the measurement model

The goodness of fit of the measurement model showed that the model had good fit with an RMSEA of 0.049 (< 0.05), and SRMR of 0.077 (< 0.08), but the CFI did not achieve the criteria of fit (CFI, 0.948). Although the χ^2^ value was significant (χ^2^ = 243.851, *df* = 179, *P* = 0.001), the χ^2^/df was < 2 (χ^2^/df = 1.362), which provided a reasonable model fit.

**Figure 1:**
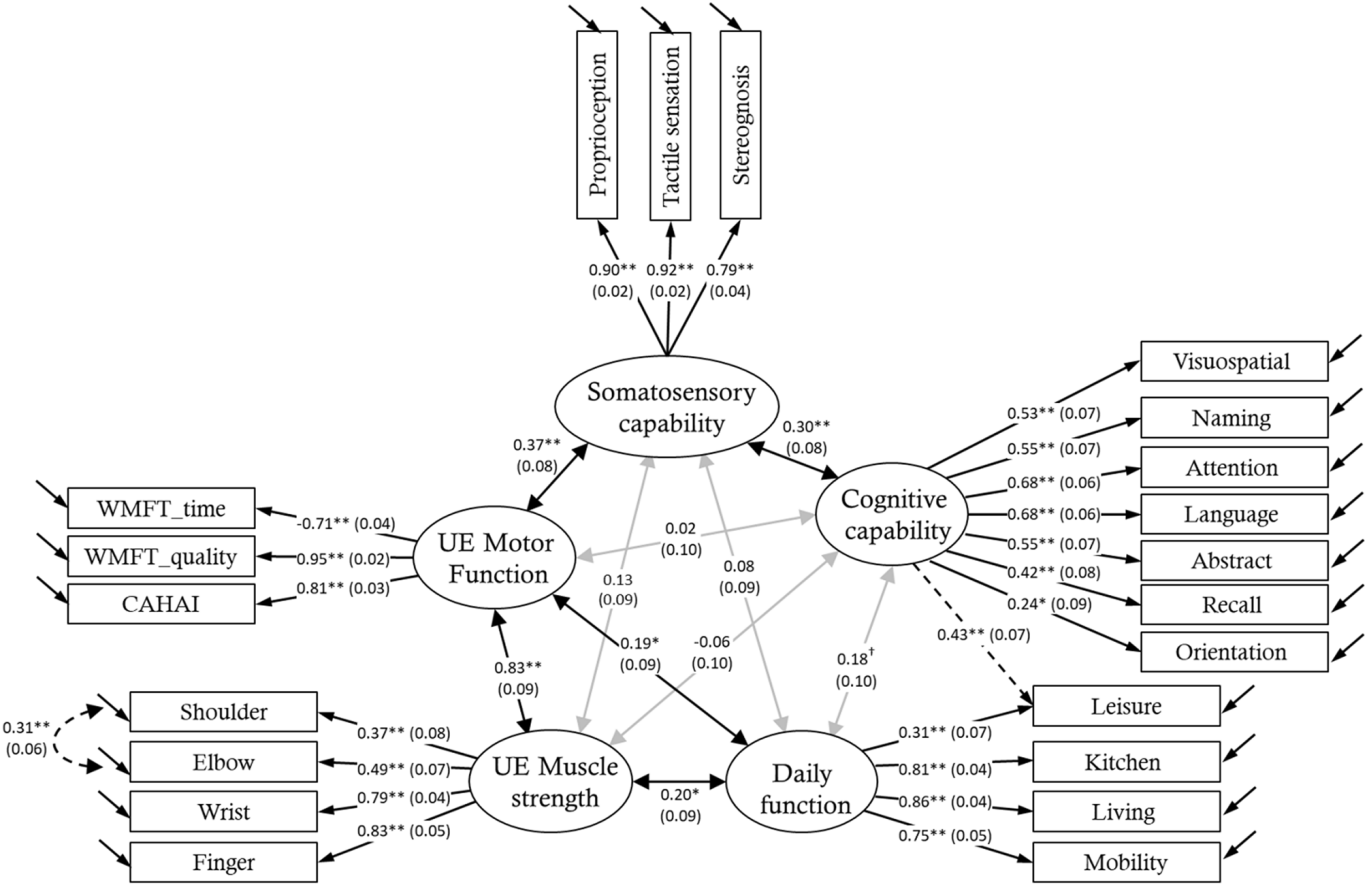
Confirmatory factor analysis of the modified measurement model. Latent constructs of five abilities (somatosensory capability, cognitive capability, UE muscle strength, UE motor function, and independence in daily function) are represented with circles and variables that measured each ability are represented with squares. Values of standardized results are shown and the standard errors are shown in the parentheses. Single-headed arrows indicate direct relationships, while double-headed arrows indicate the correlations between variables. Dark lines indicate statistically significant results, and gray lines indicate insignificant results. The dotted lines indicate the additional parameters recommended by the LM test, which indicate that the leisure activity can represent the latent constructs of both cognitive capability and independence in daily function, and measured variables of shoulder and elbow muscle are significantly correlated. ^†^*P* < 0.10; *P < 0.05; **P < 0.001. WMFT: Wolf Motor Function Test; CAHAI: Chedoke Arm and the Hand Activity Inventory.

Because the indicators did not provide consistent fitting results, we revised the measurement model based on the recommendations of the LM test to improve the goodness of fit on all indicators. We added a path from the cognitive capability to the item of leisure activity in NEADL as well as the covariance between shoulder and elbow muscle strength, and the modification index values of these 2 parameters were 23.378 and 22.323, respectively. The goodness of fit of the revised model showed a good fit (χ^2^ = 194.796, *df* = 177, *P* = 0.171; χ^2^/df = 1.101; CFI = 0.986; RMSEA = 0.026; SRMR = 0.062). Figure 1 shows the CFA results of the revised measurement model.

### SEM analysis of the structural model

**Figure 2:**
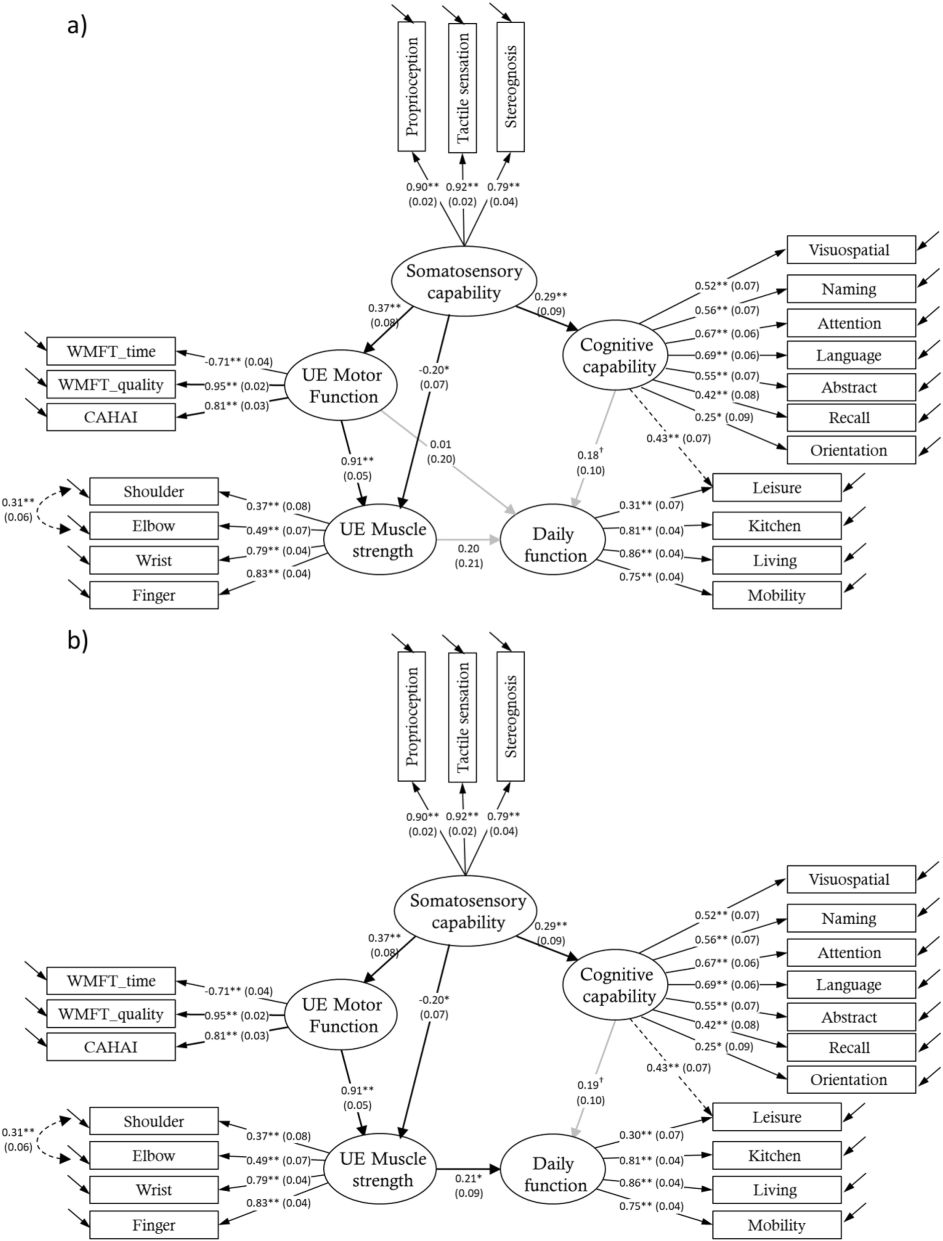
SEM analysis of the modified structural model. The values of the standardized results and the standard errors (in the parentheses) are shown. Dark arrows indicate statistically significant results, and gray arrow indicates non-significant result. (**a**) It represents the structural model revised by the recommendations of the LM test. (**b**) It represents a revised model that removed the path from the UE motor function to the independence in daily function considering the results of the Wald test and the model simplicity. ^†^*P* < 0.10; *P < 0.05; **P < 0.001.

Figure 2a describes the hypothesized model revised by the recommendations of the LM test. All fitting indexes indicated that the model showed a good fit (χ^2^ = 195.952, *df* = 180, *P* = 0.197; χ^2^/df = 1.089; CFI = 0.987; RMSEA = 0.024; SRMR = 0.064). In the standardized results, the somatosensory capability positively influenced the UE motor function performance (β = 0.368, *P* < 0.001) and the cognitive capability (β = 0.292, *P* = 0.001) but negatively influenced the UE muscle strength (β =  − 0.202, *P* = 0.004). The UE motor function showed a positive effect on the UE muscle strength (β = 0.909, *P* < 0.001). However, UE motor function, UE muscle strength, and cognitive capability had no significant effect on the independence in daily function (*P* > 0.05 for all).

To improve the precision of our hypothesized model, we used the Wald test to determine path(s) that could be reasonably removed without compromising the goodness of fit of the model. The results of the Wald test showed that 2 paths did not significantly influence the variance of the χ^2^ value (*P* > 0.06 for both). Considering that the path from cognitive capability to the independence in daily function showed a marginal but not significant effect (χ^2^ = 3.310, *df* = 2, *P* = 0.069) and that previous studies have demonstrated the important role of the cognitive capability in the daily function^19,20^, we only removed the path from UE motor function to the independence in daily function (χ^2^ = 0.002, *df* = 1, *P* = 0.961) in the model.

Figure 2b shows the path diagram of the revised model. All model-fitting indices showed good fit (χ^2^ = 195.954, *df* = 181, *P* = 0.212; χ^2^/*df* = 1.083; CFI = 0.988; RMSEA = 0.023; SRMR = 0.064). In the standardized results, the somatosensory capability positively influenced the UE motor function performance (β = 0.368, *P* < 0.001) and the cognitive capability (β = 0.292, *P* = 0.001) but negatively influenced the UE muscle strength (β =  − 0.202, *P* = 0.004). The UE motor function significantly influenced the UE muscle strength (β = 0.909, *P* < 0.001), and the UE muscle strength significantly influenced the independence in daily function (β = 0.208, *P* = 0.020).

## Discussion

To the best of our knowledge, this is the first study using SEM to demonstrate the influence of the relationship among somatosensory capability, UE muscle strength, UE motor function, and cognitive capability on the independence in daily function in chronic stroke individuals. The results (see Fig. 2b) indicate that independence in daily functions of stroke individuals would be affected by their somatosensory capability via 2 different pathways. One is mediated by their motor-related capabilities (i.e., somatosensory-motor pathway) (see the left part of Fig. 2b), and the other is mediated by their cognitive capability (i.e., somatosensory-cognitive pathway) (see the right part of Fig. 2b). Mediation effect refers to the transmission of the effect of an independent variable (i.e., somatosensory capability) on a dependent variable (i.e., independence in daily function) through other variables (i.e., UE motor function, UE muscle strength, cognitive capability)^39^.

In the somatosensory-motor pathway (see the left part of Fig. 2b), improving patients' somatosensory capability could enhance their independence in daily function either by enhancing their UE motor function performance, which in turn increased the UE muscle strength, or by directly reducing their UE muscle strength. This finding was consistent with results of previous studies that the restoration of the somatosensory capability has positive impact on the improvement of UE motor function and the independence in daily function in stroke individuals^10,11^, and UE muscle strength of the affected hand was associated with independence in daily function^6^. More importantly, the results further clarify the role of UE muscle strength in the relationship among somatosensory capability, UE motor abilities and independence in daily function, which was not mentioned in the previous research.

Regarding the role of UE muscle strength in the somatosensory-motor pathway (see the left part of Fig. 2b), intact or good somatosensory capacities might provide appropriate and sufficient somatosensory input to the brain for assisting in planning a motor output or learning a correct motor program for execution^40,41^. Accordingly, somatosensory information enhances the learning or the performance of functional use of the affected arm. Once the affected arm function improved, the stroke individuals might use the affected arm to practice a variety of tasks in a greater amount and increase the UE muscle strength. On the other hand, the pain sensations associated with the muscle spasticity and contractures^42^ may also increase with the enhancement of somatosensory capacities, thereby reducing the UE muscle strength^43^. One of the most common pain syndromes after stroke is the muscle spasticity and contractures with hemiplegia^44^. These hypertonic contractions usually present with stiffness during flexion of the upper limbs, and the prevalence of spasticity-related pain tends to peak at the chronic stage^45^. Spasticity and contractures can also cause spontaneous, painful spasms and night cramps, which can damage muscles and joints. In this case, the UE muscle strength should be reduced, because the pain sensations induced by the muscle spasticity and contractures can cause a certain degree of stiffness and tiredness in the muscles of the affected UE.

These 2 routes of the somatosensory-motor pathway (see the left part of Fig. 2b) significantly influenced the independence in daily function. However, the overall influence of somatosensory capability on UE muscle strength would be positive because the effect of the route of sensation-affected UE motor function-UE muscle strength was greater than the effect of the route of sensation-UE muscle strength.

In summary, when the ability of stroke individual’s UE motor function plays a mediation role, somatosensory capability would show positive influence on UE muscle strength; however, somatosensory capability has negative influence on the UE muscle strength. An individual’s independence in daily function is only influenced by their UE muscle strength, and UE motor function only shows the impact when it is mediated by the UE muscle strength, indicating appropriate UE muscle strength is a critical factor improving the performance of independence in daily function. Only focusing on the improvement of a patient’s functional UE performance may not guarantee the restoration of their independence in daily function.

This study identified the causal relationship between UE motor abilities and independence in daily functions, thus confirming and extending the previous findings, which only demonstrated the association between UE motor ability and the performance of independence in daily function^7^. More importantly, the current model shows that increasing the function performance of the UE should be accompanied by an improvement in its UE muscle strength. UE muscle strength thus has a key role in determining the outcome of an individual's somatosensory-motor rehabilitation.

In the somatosensory-cognitive pathway (see right part of Fig. 2b), the enhancement of stroke individuals' somatosensory ability can improve their cognitive capability, which in turn influenced their independence in daily function. Previous studies showed an association between cognitive capabilities and the performance of daily functions in stroke individuals has been demonstrated^19,20^. This study extended the previous study finding to underscore the impact of the cognitive enhancement on the independence in daily function of stroke individuals. Our finding showed that cognitive capability has a marginal contribution to the variance in the measures of the IADL performance *(P* = 0.053). It is consistent with the early findings that motor deficits had a larger impact than did perceptual and cognitive deficits on the daily function^46,47^ and may partially support the evidence that cognitive impairment does not influence functional status recovery in stroke individuals^48^.

The marginally impact of cognitive capability on the performance of independence in daily function in this study may be due to the cognitive level of the stroke individuals included and the diverse effects of different cognitive components (attention, memory, executive function, etc.) on the measurements of IADL. Given that stroke individuals preserve good general cognitive capacities, as measured by MoCA, and that independence in daily function involves familiar and routine usual activities, the patients might be capable of performing these daily tasks automatically without great involvement of cognitive processes, leading to a small impact of cognitive capabilities on the IADL performance.

On the other hand, Stephens et al.^49^ identified the differential relationships between different components of cognitive impairment (attention, executive performance, and memory) and the impairments of daily function for stroke individuals without dementia. They found that the memory deficit was not associated with any of these daily function components but that the impairments of attention and executive function were associated with disabilities in self‐care tasks and the impairment of general cognition with the impairment of complex self‐management tasks. Future research is needed to examine the impact of different cognitive domains on performance of independence in daily functions. It may provide inspiration for the development of clinical treatment methods in which differences in cognitive components are considered to design the most appropriate rehabilitation program to reestablish the independence in daily function.

The model provided by the current study shows the contribution of the somatosensory capability, cognitive capability, and UE motor abilities to the degree of the independence in daily function of stroke individuals. This model not only enhances our understanding of the mechanisms between these factors and the stroke individual’s ability to perform the independence in daily function but also might shed light on the possible directions for providing personalized treatment protocols for stroke individuals.

This study has a number of limitations. First, further studies with larger sample sizes^50,51^ and more indicators per factor^50,52^ are needed to determine the reliability and validity of this model structure.

Second, we only found a marginal effect of cognitive ability on performance of independence in daily function. As suggested by the previous findings, only specific cognitive components (i.e., attention and memory) may be critical. Future studies should be conducted to confirm the role of different cognitive components in the recovery of the independence in daily function of stroke individuals.

Finally, this study only collected the cross-sectional data on stroke individuals without severe cognitive impairment (MMSE > 20) and were younger than typical stroke populations (74.3 ± 13.6), and male participants accounted for a higher proportion of our sample^53^. The structural model may not be applicable to the entire stroke population (e.g., subacute and acute stroke, moderate to severe cognitive impairment). To increase the generalizability of the model, future studies should use data from stroke individuals with diverse characteristics.

## Conclusions

This is the first study to clarify the structural relationship among somatosensory capability, UE motor function, UE muscle strength, and cognitive capability, which play important roles in affecting the independence in daily function of stroke individuals. We found that somatosensory capability would influence the independence in daily function of stroke individuals by the somatosensory-motor pathway and the somatosensory-cognitive pathway. The former pathway suggests that somatosensory capability influences the independence in daily function through motor-related components. That is, higher somatosensory capability would decrease the UE muscle strength and increase the UE muscle strength through increasing the function use performance. Then, the variation of UE muscle strength would influence the independence in daily function of stroke individuals. The latter one indicates that somatosensory capability influences the cognitive capability, which in turn influences independence in daily function. This structural model may allow future clinical therapists to design more effective task-related training protocols to promote the independence in daily function for stroke individuals.

## Data Availability

The data that support the findings of this study are available from the corresponding author upon reasonable request. The data sharing adopted by the authors comply with the requirements of the funding institute and with institutional ethics approval.
